# Strategies for B-Cell Receptor Repertoire Analysis in Primary Immunodeficiencies: From Severe Combined Immunodeficiency to Common Variable Immunodeficiency

**DOI:** 10.3389/fimmu.2015.00157

**Published:** 2015-04-08

**Authors:** Hanna IJspeert, Marjolein Wentink, David van Zessen, Gertjan J. Driessen, Virgil A. S. H. Dalm, Martin P. van Hagen, Ingrid Pico-Knijnenburg, Erik J. Simons, Jacques J. M. van Dongen, Andrew P. Stubbs, Mirjam van der Burg

**Affiliations:** ^1^Department of Immunology, Erasmus MC, University Medical Center Rotterdam, Rotterdam, Netherlands; ^2^Department of Bioinformatics, Erasmus MC, University Medical Center Rotterdam, Rotterdam, Netherlands; ^3^Department of Pediatrics, Erasmus MC, University Medical Center Rotterdam, Rotterdam, Netherlands

**Keywords:** V(D)J recombination, next generation sequencing, CVID, immunodeficiency, repertoire

## Abstract

The antigen receptor repertoires of B- and T-cells form the basis of the adaptive immune response. The repertoires should be sufficiently diverse to recognize all possible pathogens. However, careful selection is needed to prevent responses to self or harmless antigens. Limited antigen receptor repertoire diversity leads to immunodeficiency, whereas unselected or misdirected repertoires can result in autoimmunity. The antigen receptor repertoire harbors information about abnormalities in many immunological disorders. Recent developments in next generation sequencing allow the analysis of the antigen receptor repertoire in much greater detail than ever before. Analyzing the antigen receptor repertoire in patients with mutations in genes responsible for the generation of the antigen receptor repertoire will give new insights into repertoire formation and selection. In this perspective, we describe strategies and considerations for analysis of the naive and antigen-selected B-cell repertoires in primary immunodeficiency patients with a focus on severe combined immunodeficiency and common variable immunodeficiency.

## Generation of the T- and B-Cell Repertoire

### V(D)J recombination of immunoglobulin and T-cell receptor loci

**Figure 1:**
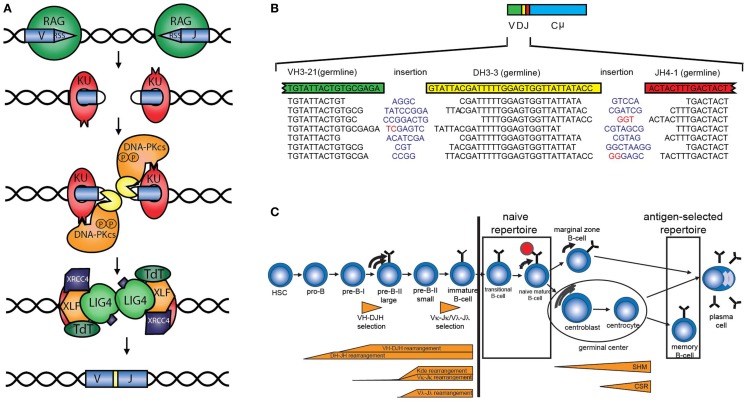
**V(D)J recombination and B-cell development**. **(A)** Schematic overview of V(D)J recombination. DNA double strand breaks are introduced by the RAG proteins, subsequently the DNA is processed and ligated by DNA repair proteins from the NHEJ pathway. **(B)** Examples of V(D)J junctions showing junctional diversity by nucleotides that are removed, non-templated (N) nucleotides (blue) that are added, or presence of palindromic (P) nucleotides (red). **(C)** The B-cell repertoire in peripheral blood can be divided into the naive repertoire and the antigen-selected repertoire.

The antigen receptor repertoire is defined as the total set of different B-cell (BR) or T-cell receptors (TRs). The loci encoding these receptors consist of multiple variable (V), diversity (D), and joining (J) genes, which can be recombined via V(D)J recombination to ensure the enormous diversity of the antigen receptors. V(D)J recombination starts with induction of DNA double strand breaks (DSBs) by the recombination-activating gene products RAG1 and RAG2 between the coding element and the recombination signal sequence (Figure 1A) (1). The DNA ends that contain the recombination signal sequence, the so-called signal ends, are blunt DNA ends, which can be ligated directly to form the signal joint. The other ends (called the coding ends because they contain the coding sequence of the Ig or TR gene) are blocked by a covalent phosphodiester bond between the top and the bottom strand of the DNA. These DNA hairpins are recognized, processed, and repaired by the non-homologous end joining pathway (NHEJ) (Figure 1A). First, the Ku70/80 heterodimer forms a ring around the DNA end that can migrate into the DNA after initial binding. Ku70/80 bound to a DNA end can then attract the DNA-dependent protein kinase catalytic subunit (DNA-PK_CS_), which acquires protein kinase activity upon DNA end binding. DNA-PK_CS_ autophosphorylation induces a conformational change in the DNA-bound complex of Ku70/80 and DNA-PK_CS_, collectively called the DNA-PK complex (2, 3). After this conformational change, Artemis opens the DNA hairpins (4, 5). If the ends are compatible, they can be ligated by ligase IV, which forms a stable complex with XRCC4. XLF (XRCC4-like factor), which has also been called “Cernunnos” (6). Before ligation, non-templated (N) nucleotides can be inserted by terminal deoxynucleotidyl transferase (TdT) or deleted via exonuclease activity (Figure 1B) (7, 8). In addition to the NHEJ components, several other factors are required to ensure efficient ligation of so-called “difficult breaks” or “complex DNA damage.” Extensive analysis of DSB repair kinetics revealed that these DSBs are mainly localized to heterochromatin and require opening of the closed chromatin structure in order to be repaired by NHEJ during the G1 phase of the cell cycle or HR in the G2 phase. Chromatin opening probably requires the initial phosphorylation of histone H2AX, the ATM kinase, the MRE11/RAD50/Nijmegen breakage syndrome (NBS)1 complex, and several enzymes necessary for ubiquitin addition near the DSB, including RNF8 and RNF168 (9–11).

### Antigen receptor repertoire diversity

The total diversity of the unique TRs and BRs is the sum of combinational diversity based on the usage of different combinations of V, (D), and J genes and junctional diversity due to nucleotide deletions by exonuclease activity, non-templated (N) nucleotide insertions that are introduced by the enzyme TdT, and the presence of palindromic (P) nucleotides that arise due to asymmetric hairpin opening by Artemis (Figure 1B). In addition, combination of the different chains (TRα and TRβ, TRγ and TRδ, or heavy and light chains) also contributes to the overall diversity. The immunoglobulin heavy chain (IGH) locus consist of 38–46 functional VH, 25 DH, and 6 JH genes resulting in a combinational diversity of >5.7 × 10^3^. In combination with the 200 possible Igκ and 124 Igλ rearrangements, this results in a combinational diversity of >1.8 × 10^6^. The total diversity of the BR is estimated to result in a total BR repertoire of >10^12^.

## Strategies for B-Cell Receptor Repertoire Analysis

### Naive versus antigen-selected B-cell receptor repertoire

The B-cell repertoire in peripheral blood can be divided into the naive and antigen-selected repertoire (Figure 1C). The naive repertoire, present in the transitional and naive mature B-cells, has not encountered antigen and most closely resembles the initial repertoire formed by V(D)J recombination during precursor B-cell differentiation. The naive repertoire can be regarded as the end-stage product of V(D)J recombination. However, it should be noted that by analyzing the naive repertoire only those B-cells can be studied that reach the naive mature B-cell stage. For studying disturbances in the V(D)J recombination itself, it can be better to analyze complete V(D)J or incomplete DJ rearrangements in bone marrow (see below). Analysis of the naive repertoire is preferably performed on DNA or RNA obtained from sorted naive B-cells. Alternatively, *IGM* transcripts can be analyzed in total RNA from peripheral blood mononuclear cells, of which only the unmutated sequences are taken into account. Usually, sequences with 98% V region identity are considered to be unmutated, since PCR and sequence errors might occur (12).

The antigen-selected repertoire is the repertoire of cells that encountered antigen, i.e., of memory B-cells. This repertoire is different from the naive repertoire, because it has undergone somatic hypermutation (SHM) with subsequent selection in the germinal center. The antigen-selected repertoire can be analyzed in sorted memory B-cells or by sequencing of *IGG* and *IGA* transcripts from RNA isolated from peripheral blood mononuclear cells.

In summary, for BR analysis, it is important to select and sort the correct B-cell population depending on the research question.

### Qualitative characteristics of the repertoire

B-cell receptor repertoire encloses a lot of information about different processes of B-cell development. The V, D, and J usage and junction composition, defined as the number of N- and P-nucleotides, deletions, and the length of the complementarity determining region (CDR)3 region, provide information about the V(D)J recombination process.

Increased numbers of P-nucleotides (13) or deletions (14), and decreased numbers of N-nucleotides are indications for a NHEJ defect (15). In addition, skewing in the usage of V and J genes can be observed, as is the case in the TR alpha repertoire in both *Xlf* knockout mice and an XLF-deficient patient (16). Vera et al. hypothesized that the reduced thymocyte lifespan does not allow the T-cells to undergo multiple waves of VαJα rearrangements that can be needed for positive selection of the T-cells. Finally, several characteristics like increased usage of certain auto-reactive VH genes, the charge of the CDR3, and increased length of the CDR3 have been associated with autoimmunity or impaired selection in patients with primary immunodeficiencies (17–20). Patients with CD19 and CD40L deficiency lack selection against long CDR3 and VH4-34, which is known to encode intrinsically self-reactive cold agglutinin antibodies that recognize carbohydrate antigens on erythrocytes (20). Similarly, a patient with RAG deficiency and autoimmunity has been described who lacked selection against these inherited auto-reactive features and in addition had skewing of the CDR3 repertoire for rearrangements with a certain CDR3 length (21).

### Productive and unproductive IGH repertoire

**Figure 2:**
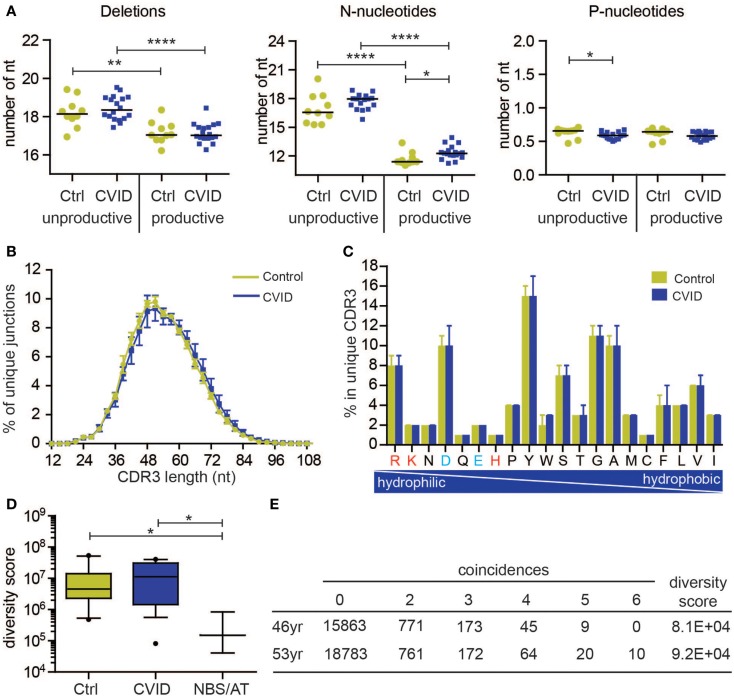
**Naive B-cell repertoire in control and CVID patients**. The naive B-cell repertoire was measured in 10 controls **(C)** and 18 CVID patients, resulting in total 293,216 unique productive rearrangements for control and 539,220 for CVID, and 127,261 unique unproductive rearrangements for control and 305,402 for CVID. **(A)** Junction characteristics of CVID patients are similar to controls. Average number of total number of deletions, N-nucleotides, and P-nucleotides are indicted per patient. **(B)** Similarly, the CDR3 length distribution (mean with SEM) of IGH rearrangements is comparable to controls. In addition, the frequency of amino acids in the CDR3 (median with range) is also comparable. The positively charged amino acids are indicated in red and the negatively charged in blue. **(D)** The diversity of the naive B-cell repertoire in CVID patients is comparable to controls, however one patient has a very restricted repertoire similar to patients with Nijmegen breakage syndrome (NBS) and ataxia telangiectasia (AT). Data are shown in box and whiskers (10–90 percentile). **(E)** The repertoire of this patient remains very restricted over time. *p < 0.05, **p < 0.01, ***p < 0.001, ****p < 0.0001.

The BR rearrangements can be amplified from DNA or from RNA. Rearrangements amplified from RNA are mostly functional (also called productive), which means that they code for a functional Ig protein. Amplification of rearrangements from DNA allows analysis of both productive and unproductive rearrangements, which have not been selected. The latter is interesting because analysis of productive and unproductive IGH rearrangements in naive B-cells in controls shows that the productive rearrangements in naive B-cells have a lower number of total N-nucleotides (13.8 versus 20.2 nt) consequently leading to a shorter CDR3 length (Figure 2A). This might be explained by the fact that in bone marrow only B-cells are selected with a shorter CDR3 region. This indicates that analysis of unproductive rearrangements could give additional information about the V(D)J recombination process and selection.

The antigen-selected repertoire is mostly studied at RNA level, by amplifying *IGG*, *IGA*, or *IGE* transcripts with primers located in or upstream of the V gene and in the constant gene. In addition to studying above mentioned characteristics of rearrangements, and SHMs can be studied (20).

### Diversity of the repertoire

For several years, conventional cloning and sequencing were the golden standard to study BR rearrangements. However, only small numbers (20–100) of rearrangements could be studied, which give a limited overview on the diversity of the total repertoire. Therefore, the current next generation sequencing (NGS) approaches, which enable to study thousands of rearrangements, give new opportunities for studying the diversity of the repertoire. Although the potential BR repertoire is estimated to be over 10^12^, this number exceeds the total number of different B-cells present in an individual. When analyzing the diversity of the repertoire by NGS, only a small fraction of the total B-cell population is analyzed. When studying the antigen receptor repertoire diversity, it is of great importance to take into account the number of B-cells that was used to generate the repertoire data. If you start with 100,000 B-cells, one cannot expect to find more than 100,000 different productive rearrangements. Since every B-cell contains only one DNA copy of a productive rearrangement, the total number of productive rearrangement can be estimated when using a fixed DNA input of sorted B-cells. Every cell contains around 6 pg of DNA, so 100 ng DNA represent approximately 16,667 cells, which also equals the number of productive rearrangements that can be expected. In general, every B-cell contains only one productive rearrangement, however multiple B-cells with the same rearrangements can be present in the B-cell pool being studied.

When using RNA for the repertoire analysis it is difficult to estimate the number of cells, because every B-cell contains multiple RNA copies of the same rearrangement, and plasma cells contain more RNA copies of the rearrangement compared to other B-cell subsets. So, when multiple copies of the same rearrangement are found, these can be derived from the same B-cell, two independent B-cells, or they can be a technical duplication. One should therefore be careful interpreting the data, and for repertoire calculations, it is best to only take the unique productive rearrangements in to account, unless randomly barcoded primers to identify individual B-cells are used (22).

In literature, different methods for diversity calculations are used. Species richness and Shannon entropy are frequently used methods (23, 24). The Species richness takes into account the number of different rearrangements in a sample, whereas the Shannon entropy also takes the frequency of the rearrangement into account (23). The latter methods are usually performed on rearrangements derived from an individual PCR amplification. Boyd et al. used a slightly different method in which occurrences of rearrangements between six individual PCR within the same individual was measured (25). These occurrences, also called coincidences, could only be derived from clonally related B-cells. Immunocompetent individuals should have little coincidences since they have a diverse repertoire, while immunocompromised individuals are likely to have more coincidences since they have a more restricted repertoire.

## B-Cell Receptor Repertoire Analysis in SCID

In patients with primary immunodeficiencies, antigen receptor repertoire formation or selection can be disturbed, resulting in restricted antigen receptor repertoire diversity. Depending on the genetic defect, this can result in severe immunodeficiency as is the case in severe combined immunodeficiency (SCID) patients with defects in RAG1/RAG2 or in components of the NHEJ pathway with severely impaired V(D)J recombination (6, 14, 26–29). These genetic defects not only result in a quantitative V(D)J recombination defect, but also in a qualitative defect. Because of the qualitative V(D)J recombination defects, there are no peripheral B-cells or the number of B-cells is too low for reliable analysis. In this case, the repertoire can be studied by analyzing incomplete or complete BR rearrangements in bone marrow-derived precursor B-cells. NHEJ defects leads to aberrant formation of V(D)J junctions and greatly impact junctional diversity, which implies that NHEJ defects cause a quantitative and qualitative antigen receptor repertoire defect. For example, mutations in *Artemis* and *DNA-PKcs* (13, 29), which affect hairpin opening, result in a significant increase in the number of P-nucleotides, whereas defects in *XRCC4* result in significantly reduced numbers of N-nucleotide insertions (15).

Also, other genetic defects, like ATM and NBS deficiency, result in reduced diversity of the naive B-cell repertoire. These proteins are not directly involved in the V(D)J recombination process itself, but are important for sensing of the DNA DSBs and for keeping the two DNA ends together during V(D)J recombination. As the result of less efficient V(D)J recombination, patients have reduced numbers of naive B-cells in the periphery, which can in addition show an increased proliferation history which results in even more restriction of the repertoire. This is what we have shown in patients with mutations in *ATM* causing ataxia telangiectasia (AT), which is involved in DSB sensing and juxtaposition of DNA ends, impair antigen receptor repertoire diversity (30). Similar observations have been done in patients with NBS (31).

## B-Cell Receptor Repertoire Analysis in CVID

### No qualitative defect in the naive IGH repertoire of CVID patients

Common variable immunodeficiency (CVID) is the most common primary antibody deficiency characterized by hypogammaglobulinemia and poor response to vaccination resulting in infections and in some patients to non-infectious complications including autoimmunity, lymphoproliferative disease, malignancies, and granulomas. In the majority of patients (95%), the genetic defect is unknown, but several studies have demonstrated defects in B-cell differentiation. Therefore, CVID patients could have a restricted BR repertoire underlying the disease.

Driessen et al. showed that a subgroup of CVID patients also have a reduced number of naive B-cells, with an increased proliferation history, like the AT and NBS patients. (32) Therefore, we hypothesized that a subgroup of patients with CVID might have a reduction in the diversity of the naive IGH repertoire, possibly caused by less efficient V(D)J recombination. We studied the naive IGH repertoire in 18 patients described by Driessen et al. (32) and 10 healthy controls. We sorted naive B-cells and amplified the VH–JH junctions in six independent PCRs using 100 ng DNA per PCR. For the analysis of the qualitative parameters, we combined the rearrangements of all six individual PCRs and selected the productive or unproductive unique rearrangements based on the V and J gene and the amino acid sequence of the CDR3.

All of the qualitative parameters we studied were similar to healthy controls in this group of CVID patients. The composition of the junctions in both productive and unproductive rearrangements was normal (Figure 2A). The only differences were a slightly higher number of N-nucleotides (average difference of 0.7 nucleotides) in productive rearrangements, and a very small decrease (on average 0.04 nucleotides) in the number of P-nucleotides in unproductive rearrangements. However, this did not result in a shorter CDR3 length (Figure 2B). Similar to controls, the productive rearrangements had significantly less deletions and N-nucleotide insertions, indicating selection for shorter CDR3 length. Furthermore, the CDR3 length distribution and composition of hydrophilic and hydrophobic amino acids was also similar to controls (Figures 2B,C). These data suggest that these CVID patients do not have a qualitative defect in the naive repertoire.

### Diversity of the naive IGH repertoire is normal in most CVID patients

To measure the diversity of the naive IGH repertoire, we used the method proposed by Boyd et al. (25). Based on the occurrence of rearrangements between six independent PCRs, the diversity of the repertoire can be calculated. We expressed the diversity of the repertoire in a diversity score, in which a low value indicate a restricted repertoire and a high value represents a more diverse repertoire. Both in the control as in the CVID patients, there is some spread in the diversity of the repertoire (Figure 2D). However, one CVID patient clearly had a more restricted repertoire that the controls. This repertoire was as restricted as patients with NBS and AT deficiency (Figure 2D). To assess if this reduction in the repertoire is stable over time, the repertoire was analyzed at a second time point 7 years later (Figure 2E). At both 46 and 53 years of age, this patient had a very restricted repertoire. At the second time point, there were even 10 rearrangements present in all individual PCRs, indicating that in the small sample of blood that we took, at least six B-cells had the same IGH rearrangement. Interestingly, this patient has a family history of breast cancer indicative for a possible DNA repair defect. These data show that patients with a restricted repertoire can be identified using this method, however, most CVID patients have a very diverse naive repertoire. Based on the B-cell patterns identified by Driessen et al., it is expected that most of the CVID patients will have problems after the naive B-cell stage. It will therefore be very interesting to also study the antigen-selected repertoire in patients with CVID.

## Future Perspectives

Developments in NGS give possibilities to study the antigen receptor repertoire of patients at a very detailed level than ever before. Different strategies can be followed to address many research questions related to the pathophysiology of primary immunodeficiency (PID). In this perspective, we focused on SCID and CVID, but it has already been proven valuable for many other PIDs (33). The next challenge will be linking antigen receptor repertoire data to antigen reactivity and specificity, which might in future be linked to specific clinical features.

## Methods

### Repertoire analysis using next generation sequencing

Naive B-cells were sorted from peripheral blood from 18 CVID patients and 10 healthy controls. DNA was isolated using direct lysis and IGH rearrangements were amplified and sequenced using Roche 454 sequencing as previously described (30). In short, IGH rearrangements were amplified from a multiplex PCR using the forward VH1-6 FR1 and reverse JH consensus BIOMED-2 primers (34). PCR products were purified by gel extraction (Qiagen, Valencia, CA, USA) and Agencourt AMPure XP beads (Beckman Coulter). Subsequently, the PCR concentration was measured using the Quant-it Picogreen dsDNA assay (Invitrogen, Carlsbad, CA, USA). The purified PCR products were sequenced on the 454 GS junior instrument according the manufacturer’s recommendations. Sequences were demultiplexed based on their multiplex identifier sequence and trimmed using the IGGalaxy tool (35). Fasta files were uploaded in IMGT/High-V-Quest (36), and subsequently, the IMGT output files were analyzed in the IGGalaxy tool (35). Information on junction characteristics, CDR3 length, and composition were extracted from the data provided by IMGT. The repertoire diversity score was calculated by dividing 1 to the clonality score, which is given by the IGGalaxy tool and is based on the calculation of Boyd et al. (25).

### Statistics

Significance differences were calculated using the two-tailed Mann–Whitney test in the GraphPad Prism program (GraphPad Software, Inc.).

## Conflict of Interest Statement

The authors declare that the research was conducted in the absence of any commercial or financial relationships that could be construed as a potential conflict of interest.
